# Locally adapted populations of a copepod can evolve different gene expression patterns under the same environmental pressures

**DOI:** 10.1002/ece3.3016

**Published:** 2017-05-09

**Authors:** Thiago G. Lima, Christopher S. Willett

**Affiliations:** ^1^ Department of BiologyUniversity of North Carolina at Chapel HillChapel HillNCUSA; ^2^ Present address: Marine Biology Research DivisionScripps Institution of OceanographyLa JollaCAUSA

**Keywords:** local adaptation, phenotypic plasticity, RNA‐seq, *Tigriopus californicus*

## Abstract

As populations diverge in allopatry, but under similar thermal conditions, do similar thermal performance phenotypes evolve by maintaining similar gene expression patterns, or does genetic divergence lead to divergent patterns of gene expression between these populations? We used genetically divergent populations of the copepod *Tigriopus californicus*, whose performance at different thermal conditions is well characterized, to investigate transcriptome‐wide expression responses under two different thermal regimes: (1) a nonvariable temperature regime and (2) a regime with variable temperature. Our results show the expression profiles of the response to these regimes differed substantially among populations, even for populations that are geographically close. This pattern was accentuated when populations were raised in the variable temperature environment. Less heat‐tolerant populations mounted strong but divergent responses to the different thermal regimes, with a large heat‐shock response observed in one population, and an apparent reduction in the expression of genes involved in basic cellular processes in the other. Our results suggest that as populations diverge in allopatry, they may evolve starkly different responses to changes in temperature, at the gene expression level, while maintaining similar thermal performance phenotypes.

## 1 Introduction

Understanding how ectotherms adjust to changing environments across generations, through adaptation, or within a generation, through phenotypic plasticity, is issue that has been of interest to evolutionary biologists and physiologists for some time. These processes can be a driver of divergence and represent a key component of fitness for many organisms (Angilletta, 2009). More recently, studies in this area have also become important as a way to predict how well taxa will fare given rates of global change, especially for organisms with limited potential for dispersal (Hoffmann & Sgrò, 2011). One interesting question is the degree to which independently evolving populations will begin to diverge in their genetic response to thermal challenges, that is, will they develop different solutions to the same problem?

Early studies of thermal adaptation revealed the importance of heat‐shock proteins (HSPs) as one of the mechanisms to protect the cell from damage following exposure to stressful conditions (Feder & Hofmann, 1999; Lindquist, 1986), and now more recent results from transcriptomic studies are highlighting a wider variety of genes important for thermal response (DeBiasse & Kelly, 2016). The genes coding for HSPs are often found to be upregulated in a range of organism under thermal stress (Tomanek & Somero, 1999; Sorensen et al., 2005; Bedulina, Zimmer, & Timofeyev, 2010; Schoville et al., 2012; Barshis et al., 2013; Wang et al., 2014; Gleason & Burton, 2015 but exceptions do exist: e.g., Franssen et al., 2014). The advent of next‐generation sequencing has allowed for transcriptome‐wide studies in nonmodel organisms, which has improved our knowledge of the genetic basis of thermal adaptation and plasticity beyond what is known from candidate genes (reviewed in DeBiasse & Kelly, 2016). Besides the upregulation of HSP genes, other common responses to thermal stress have also been described; these responses included an overrepresentation of genes with Gene Ontology terms associated with the production of molecules that prevent cell damage from reactive oxygen species, molecules that target proteins that have been damaged beyond repair for proteolysis, immune‐system related genes, and cell wall/membrane modification genes (Barshis et al., 2013; Dayan, Crawford, & Oleksiak, 2015; Franssen et al., 2014; Gleason & Burton, 2015; Narum & Campbell, 2015; Wang et al., 2014). While the functions of these genes are similar across studies, there is a lot of variation in the set of genes that respond to thermal stress. This is in part because of differences in study design, but also because these divergent taxa have evolved different ways to deal with thermal changes. Therefore, we were interested in determining how much the transcriptome‐wide response varies when divergent populations of the same species are compared under different thermal regimes. As populations diverge in similar environments but in allopatry, does selection favor similar genetic responses across the populations? Or do novel mutations that appear in each population contribute to increased divergence in their response to thermal changes?

**Figure 1:**
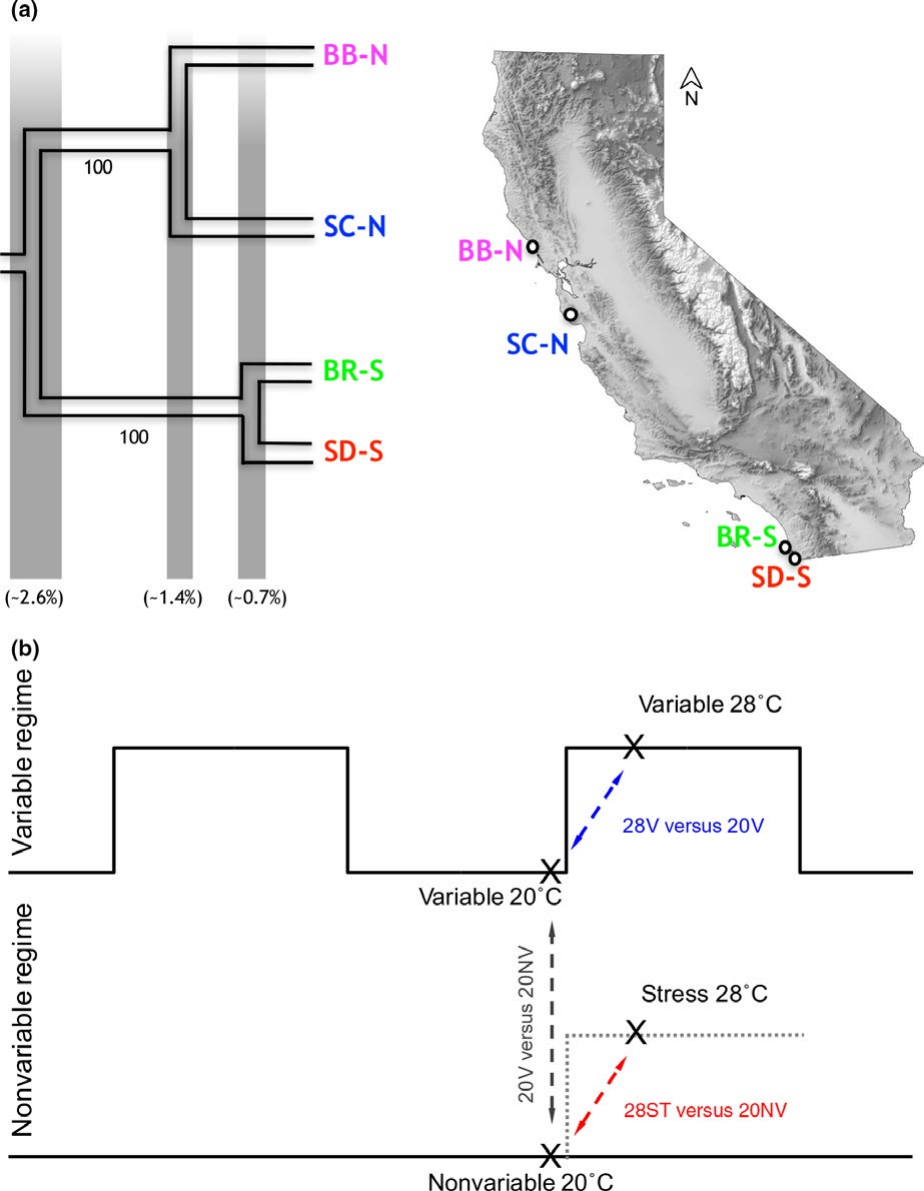
Phylogeography of populations and experimental design. (a) Phylogeny and sampling locations for the four populations in this study. Branch lengths reflect average genetic divergence. Numbers below branches are bootstrap support. Percentages in parentheses are averages of uncorrected divergence based on 11,560 nuclear loci (figure modified from Pereira et al., 2016); (b) populations were exposed to two thermal regimes (nonvariable and variable). Gene expression was assayed from both regimes at 20° at the end of the 20° portion of the variable regime. Copepods from the nonvariable regime were moved to 28° at this point. RNA was isolated from both regimes at 28°, after two hours at this temperature. Dashed arrows indicate pairwise comparisons that were made to calculate relative gene expression between treatments. In red: nonvariable at 20° (20NV) compared to stress at 28° (28ST); in blue: variable at 20° (20V) compared to variable at 28° (28V); in dark gray: 20NV compared to 20V

The copepod *Tigriopus californicus* is an ideal species in which to test this, as it gives us the opportunity to compare a number of locally adapted populations across a broad latitudinal spectrum differing in thermal tolerance. Populations of this species inhabit splash pools on the Pacific coast of North America and can be exposed to large changes in temperature on a daily basis. Gene flow is extremely low, which has led to the formation of many genetically divergent populations, even for populations that are only a few kilometers apart (Burton, 1997; Willett & Ladner, 2009). Within California, northern and southern populations fall out into two clades, with the northern clade encompassing populations north to Alaska (Burton, 1998; Edmands, 2001; Willett & Ladner, 2009; Figure 1a). Populations from the two clades show significant differences in local adaptation, particularly for thermal tolerance where upper thermal tolerance increases as latitude decreases, with lethal temperatures ranging from 35° in the northern clade to 38° in the southern clade (Kelly, Sanford, & Grosberg, 2012; Pereira, Barreto, & Burton, 2014; Tangwancharoen & Burton, 2014; Willett, 2010). In a study of acute thermal stress (35°), Schoville et al. (2012) found that *T. californicus* from a southern clade population (San Diego, CA) showed much greater upregulation of genes that are known to respond to heat stress, such as heat‐shock proteins, than did individuals from a northern clade population (Santa Cruz, CA), suggesting that their ability to upregulate these genes may be at least in part responsible for their higher heat tolerance. The northern population differentially expressed a much higher number of genes overall (both up‐ and downregulated), but to much lower levels of fold change.

Differences in thermal adaptation in this species can also be observed as a shift in the thermal performance curve (TPC) of populations from south to north (Hong & Shurin, 2015). The width of the TPC remains the same, but the fitness peak shifts from warmer to cooler temperatures as you move north. In agreement with this, Willett (2010) showed that at higher nonlethal daily variable temperatures (20°–28°), two different southern populations outcompete two other northern ones, and the opposite is true for colder variable temperatures (16°–25°) or lower nonvariable temperatures (16°). The populations have roughly equivalent fitness at 20° (Willett, 2010). Both southern and northern populations commonly experience temperatures to and beyond 28° in nature, and when in the high variable temperature environment by themselves, all populations are able to develop and have offspring normally (Willett, 2010). Therefore, it seems northern populations are unable to survive in these high temperatures because of low competitiveness compared to the more heat‐tolerant southern populations.

In contrast to numerous studies carried out at high, nearly lethal stressful temperatures, fewer studies of genetic response have been carried out under moderate and variable temperatures that many organisms may encounter on a regular basis (but see Barshis et al., 2013; Kenkel, Meyer, & Matz, 2013; Franssen et al., 2014; Dayan et al., 2015; Kenkel & Matz, 2016). Here, we look at transcriptome‐wide gene expression under such moderately stressful temperatures in different populations of *T. californicus*. This study has two aims: (1) to determine how similar the transcriptome‐wide response is between relatively geographically proximate populations with similar thermal phenotypes (the two northern and two southern populations used in Willett, 2010) and (2) to elucidate the molecular responses that underlie the ability of copepods from these southern populations to outcompete the northern ones at higher, variable temperatures but not at lower temperatures (Willett, 2010). We use two different thermal regimes, with a total of four treatments, and compare the transcriptome‐wide response between the four populations from Willett (2010). By comparing the response from each population, we get a picture of how each population has adapted to deal with these temperature changes; while comparisons between treatments within populations show us the plastic response, these populations are able to mount given changes in temperature. The combined results show that the transcriptome‐wide response from each population differs greatly even between populations that are only 8 km apart (the two southern populations) and is even greater between the northern populations. The difference in gene expression changes also increased when organisms were raised in the variable temperature regime, compared to a nonvariable regime followed by a one‐time moderate heat stress. Our results suggest that the less heat‐tolerant northern populations are potentially outcompeted by southern ones at least in part due to the costs associated with changing the expression of a much higher number of genes when in the moderately high variable temperature regime.

## 2 Materials and Methods

### 2.1 Copepod collection, culture, and thermal treatments

Copepods were collected in May 2013 from rocky intertidal pools at four sites, the two southern populations of San Diego (SD‐S, 32°44′44″N, 117°15′18″W) and Bird Rock (BR‐S, 32°48′54″N, 117°16′23″W), and the two northern populations of Santa Cruz (SC‐N, 36°56′58″N, 122°02′49″W) and Bodega Bay (BB‐N, 38°19′4″N, 123°4′23″W) (Figure 1a). Copepods were maintained *en masse* cultures in petri dishes in 35 ppt artificial seawater (Instant Ocean, Aquarium Systems) and consumed both commercial fish food and natural algae growth. Cultures were kept in incubators with 12‐hr light:dark cycle at 20° for two to three generations before beginning the experiment. Copepods were exposed to two thermal regimes: (1) a nonvariable 20° and (2) a variable environment with 12 hr at 20° and 12 hr at 28° each day. Although the variable environment is more similar to the natural environment where daily temperature fluctuations are common, aspects such as rate of temperature change may be less representative (but are identical to those used in the Willett (2010) study). For both regimes for all populations, 25 gravid females were transferred to petri dishes (four dishes per population per treatment) and randomly assigned to one of the thermal regimes. Mothers were removed when offspring reached the copepodid stage. Therefore, copepods were born in their respective thermal regimes and stayed in this regime until they reached the adult life stage (copepodid stage 6) at which point RNA was extract for the different treatments. Differences in gene expression for each population, between thermal regimes, should be due to plasticity differences and not adaptation, as the copepods were in the different regimes only for the one generation. On the day before RNA isolation, 100 adult copepods (50 males and 50 females) were transferred to a new petri dish, without additional food. Each petri dish with 100 individuals was a replicate for one of the treatments. For each treatment, all populations had two dishes with 100 copepods and a separate mRNA library was created from the pool of 100 copepods for each population/treatment replicate (Fig. S1). As shown in Figure 1b, there were two sampling points for each thermal regime: (1) At the end of the 20° period in the variable regime, RNA was extracted from copepods from both regimes (20NV and 20V); at this point, copepods from the nonvariable regime were transferred to 28°; (2) 2 hr after the temperature shift to 28° on the variable regime, RNA was extracted from copepods at 28° from both regimes (28ST and 28V) (Figures 1b; S1.).

### 2.2 RNA extraction and Illumina sequencing

For each treatment at the appropriate sampling time, copepods from each dish (~100 individuals) were collected onto filter paper, rinsed with filtered seawater, and immediately transferred to 250 μl of TRI Reagent (Sigma). RNA isolation was performed following the manufacturer's protocol. RNA samples were quantified using Qubit 2.0 fluorometer (Life Technologies), and 2 μg of total RNA per sample were submitted to the UNC High‐Throughput Sequencing Facility for library preparation and sequencing. RNA‐seq libraries were prepared using the TruSeq Sample Prep Kit (Illumina), and samples were sequenced as 100‐bp single‐end unstranded mRNA libraries in the Illumina HiSeq 2000 (for one replicate of SD‐S and SC‐N, and both replicates of BR‐S and BB‐N) and HiSeq 2500 (for the second replicate of SD‐S and SC‐N). Each Illumina sequencing lane contained two populations (SD‐S and SC‐N; BR‐S and BB‐N), with all treatments barcoded. Replicates were sequenced in the same manner in separate lanes, for a total of four lanes. A total of 32 libraries were sequenced (four populations x two thermal regimes x two treatments per thermal regime × two replicates per treatment). Illumina reads were deposited in the NCBI SRA BioProject (PRJNA308869). Illumina reads were trimmed for quality using CLC Genomics Workbench (CLC GW) 7.0.4 (CLC Bio) discarding bases with Phred score lower than 20, and keeping reads with at least 15 bp remaining after trimming.

### 2.3 Transcriptome assembly and annotation

An annotated reference genome for the SD‐S population has been published online (https://i5k.nal.usda.gov/Tigriopus_californicus), and it was used to build transcriptome references for all populations in this study as follows. Annotated genes from the SD‐S reference genome were extracted, and alternative splice variants were removed, keeping only the longest splice variant of a gene. To avoid a bias in mapping of reads from SD‐S to its own high‐quality reference with reads from the other populations likely to have some SNP differences, we built references from each of the four populations by mapping them to the SD‐S genes. Trimmed reads from all treatments were combined for each single population and mapped to the SD‐S genes using BWA MEM using default parameters (Li & Durbin, 2009). Following mapping, reads with low mapping quality (MAPQ < 20, likely the result of incorrect alignment) were removed, and the consensus sequences for each population were extracted using SAMtools and BCFtools (Li, 2011; Li et al., 2009). We assessed the quality of the references by calculating the total percentage “N” in the reference transcript set of each population. We also compared the sizes of the orthologous contigs and the percentage “N” in each contig for all transcripts that occurred in all four populations. As our gene expression analysis only compared within‐population differences in treatment reads mapped to their own reference, no further action was taken at this stage of the analysis to remove short transcripts with a high percentage of “N,” as these were removed during gene expression analysis.

The current SD‐S genome assembly is thought to include ~80% of the entire genome, and certain genes are known to not be in this reference. To avoid excluding these genes from our analysis, we used de novo transcriptome assemblies from Pereira et al. (2016) to complement the pool of genes in our analysis as the populations used in this study are the same as the ones used in Pereira et al. (2016). To avoid confusion, we will refer to the set of genes relating to the reference genome as genomic transcripts (GTs), and from the de novo transcriptome assembly as de novo transcripts (DNTs). Transcripts originating from the different references can be differentiated based on their ID, where GT IDs begin with “TCALIF,” while DNT IDs begin with either “Contig” or “comp.”

BLAST2GO (Conesa et al., 2005) was used to annotate transcripts from the references. We used the SD‐S reference for annotation as it had slightly higher quality than the others. BLASTX searches were performed against the “nr” NCBI protein database, retaining hits with *E ≤ *10^−3^. Gene Ontology (GO) terms (Ashburner et al. 2000) were retrieved for contigs with positive BLAST hits, with an *E ≤ *10^−3^.

### 2.4 Mapping and identification of differentially expressed genes

Trimmed reads for each treatment were mapped to their respective population's GT references in CLC GW (mapping parameters: similarity fraction = 0.99; length fraction = 0.8; mismatch cost = 2; insertion cost = 3; deletion cost = 3). Unmapped reads were retained and mapped to the DNT references using the same mapping parameters. Only unique mapped reads from the two sets of mapping files were used in further analyses. Only orthologous genes that occurred in all four populations were considered. Orthologous genes from the GT reference already had the same ID, while those from the DNT reference were extracted from the orthologous list in Pereira et al. (2016) and were given the SD‐S ID for ease of comparison in later steps.

Differential expression was determined using the Bioconductor package edgeR (Robinson, McCarthy, & Smyth, 2010), in pairwise comparisons between treatments within each population. As the pairwise comparisons were not simply “control versus treatment,” separate files for each pairwise comparison for each population were created and analyzed separately. Genes with very low levels of expression in both treatments were removed by retaining only those that accumulated at least two counts per million in at least two of four samples, allowing for both replicates of a treatment to have zero mapped reads, if both replicates in the other treatments had enough reads mapped to them. This also removed all genes that had zero mapped reads in the DNT reference, because the appropriate reads for these transcripts had already mapped to the GT reference. Compositional differences between the libraries were normalized using the trimmed mean of M‐value method (Robinson & Oshlack, 2010). For each population, all treatments were examined for possible batch effects between the replicates, using a multidimensional scaling (MDS) plot to visualize the level of similarity between each RNA sample for each population (Fig. S2). To account for batch effects between the replicates, a negative binomial generalized linear model (GLM) was fit, where “sequencing lane” was used as a blocking factor. Likelihood ratio tests were used to determine genewise expression differences between two treatments, followed by a false discovery rate (FDR) correction of *p* values set to 5% (Benjamini & Hochberg, 1995). Genes that were detected as differentially expressed (DE), but had 0 reads mapped to both replicates in one of the treatments, were considered DE but were excluded in comparisons of the magnitude of their relative expression, as they showed abnormally high levels of fold change. Gene expression data are available through the NCBI Gene Expression Omnibus under Accession No. GSE80737.

### 2.5 Pairwise comparisons of relative gene expression

Three pairwise comparisons between treatments were performed in EdgeR, each aimed at answering a specific question (Figure 1b). To determine how populations respond to potentially low levels of thermal stress, relative expression between the nonvariable 20° (20NV) and stress 28° (28ST) treatments was calculated. To determine how populations respond to daily fluctuations in temperature, relative expression between the variable 20° (20V) and variable 28° (28V) was calculated. Differences between these two comparisons (28ST vs. 20NV vs. 28V vs. 20V) should then reflect the effects of phenotypic plasticity responses to exposure to 28°. To determine whether genes were being differentially expressed between variable and nonvariable regimes, relative expression between 20NV and 20V was calculated.

### 2.6 Gene ontology enrichment analysis

Enrichment of Gene Ontology (GO) terms was assessed in Blast2GO using Fisher's exact tests for every GO term that appeared in a subset of genes, compared to all genes used in the analysis for each population (reference set). *p‐*values were adjusted for multiple comparisons using a FDR set at 5%. GO enrichment analysis was performed for up‐ and downregulated genes separately for each pairwise comparison in each population. In many cases, redundant GO terms were significantly enriched in a dataset, in which case only one of these terms was included in our results using the following criteria: (1) If the terms associated with the same transcript belonged to different GO categories (i.e., biological process (P), molecular function (F), or cellular component (C)), they were all kept; (2) the most specific term was kept if it included approximately the same number of genes as the more broad term (e.g., response to stress (more specific) had 13 genes, response to stimulus (broader) also had 13 genes, response to stress was kept); (3) when multiple more specific terms combined included most of the genes in their common broader term, the specific ones were kept.

## 3 Results

### 3.1 Illumina sequencing and RNA‐seq mapping

RNA sequencing yielded ~7.6–29 million reads per sample after trimming (Table S1). The genomic transcript (GT) references created by mapping RNA‐seq reads to the annotated *T. californicus* reference genome yielded 13,839 orthologous transcripts, while the de novo transcript assembly (DNT) from Pereira et al. (2016) included 12,576 orthologous transcripts, for a total of 26,415 transcripts. Of these, 20,211 (76%) had a significant BLAST hit and were assigned a gene name. GO terms were retrieved for 17,422 transcripts (66%). The approximately 26,000 transcripts is an overestimate of the number that factored in the analyses for two reasons. First, the two transcriptomes are redundant (redundant DNT transcripts were dropped after mapping reads as described in the methods), and second, several transcripts were filtered out during differential expression analysis due to low number of mapped reads. The resulting datasets varied slightly for each population because the transcripts with low coverage were not always the same, but all genes included in any of the populations’ set occur in all other populations, even if they were dropped due to low number of mapped reads in this study. The number of transcripts used for the remainder of the study was as follows: SD‐S: 18,471 (11,715 GT, 6,756 DNT); BR‐S: 18,018 (11,676 GT, 6,342 DNT); SC‐N: 18,962 (11,573 GT, 7,389 DNT); BB‐N: 18,294 (11,516 GT, 6,778 DNT).

The MDS plot showed that all RNA samples clustered strongly by population (Fig. S2a). Within each population, treatments and replicates showed some separation based on the sequencing lane they were in, but there was also a lack of consistent grouping between replicates of the same treatment (Fig. S2b‐e). A likely explanation for this is the fact that our treatment temperatures were not extreme, 28° is only moderately stressful to these populations, and are not expected to cause large consistent changes in expression for the large majority of the transcriptome. RNA was extracted from a pool of 100 individuals for each treatment, and only a small percentage of all the transcriptome is responding in the same way across all tissues of all individuals. However, this should not be seen as negative outcome; as suggested by Sorensen et al. (2005), this averaging across tissues is an advantage as we are able to identify general responses taking place in several tissues and across several individuals. This should increase our power to detect genes that are globally important for thermal regulation.

### 3.2 Response to moderate thermal stress in the two thermal regimes

When the different populations experience 28°, we expect northern populations to be more stressed as they have lower heat tolerance and their thermal performance curve is shifted to colder temperatures. Therefore, in both thermal regimes [nonvariable (NV) and variable (V)], we hypothesized that the northern populations will have to mount a stronger heat‐shock response, which should translate into a larger number of differentially expressed (DE) genes, as well as higher magnitude of change for these genes. We expect, especially in northern populations, upregulated genes to be involved in the heat‐shock response pathway (such as HSP genes), but not necessarily upregulation of genes associated with proteolysis or apoptosis as the high‐temperature treatments are well below the populations’ lethal temperatures. We also expect to see the downregulation of genes associated with normal cell maintenance as the heat‐shock response is known to favor the synthesis of HSPs while suppressing the expression of other genes (Tomanek, 2010). Southern and northern populations are expected to share more DE genes when compared to the population in the same region, as opposed to comparisons between regions. Our results, however, did not support (or only partially supported) our expectations. Below we detail the results for each thermal regime comparison.

### 3.3 Response to moderate thermal stress after nonvariable temperature regime (28ST vs. 20NV)

**Figure 2:**
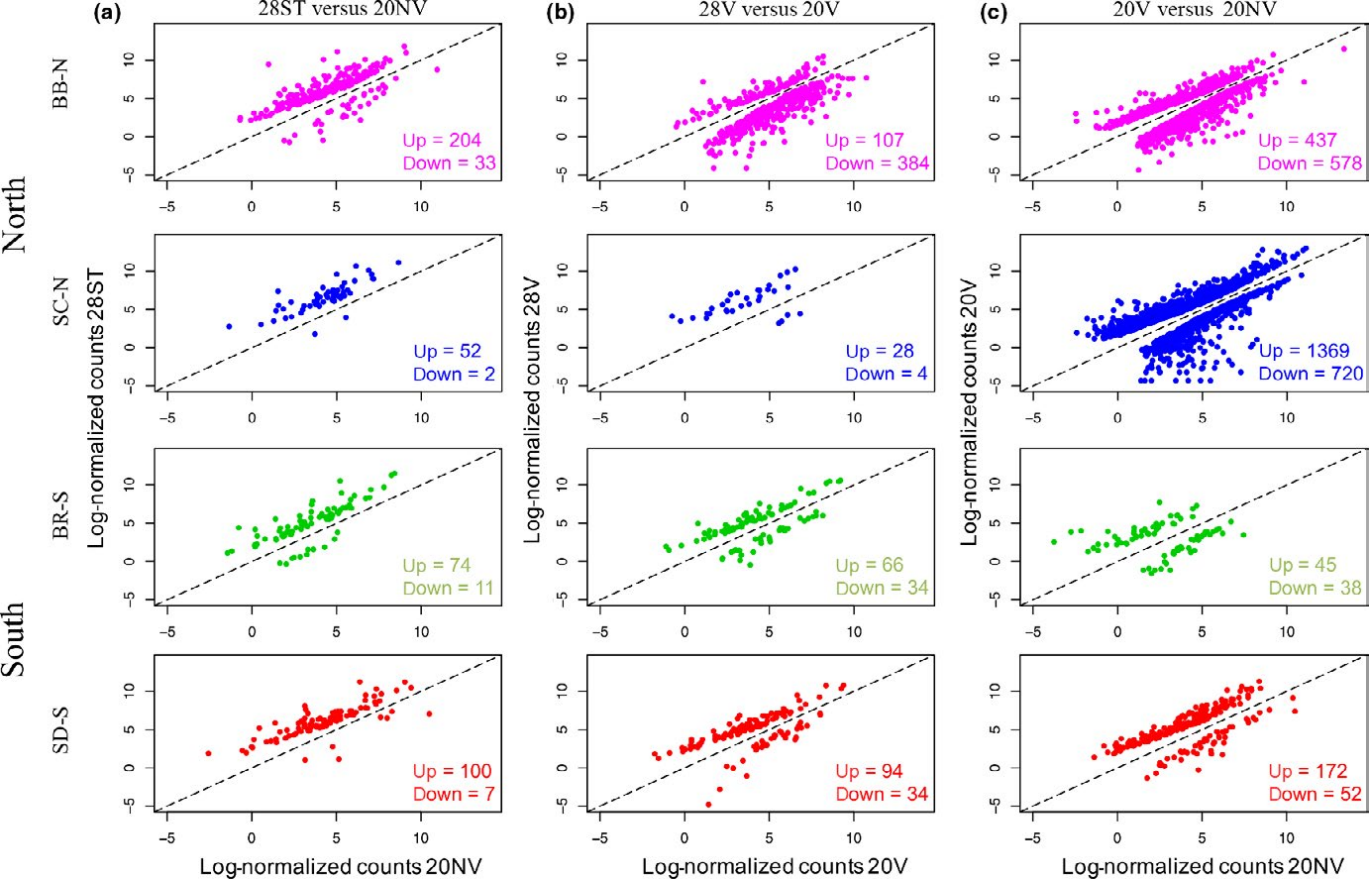
Differentially expressed genes for the three pairwise treatment comparisons. (a) Stress 28° (28ST) versus nonvariable 20° (20NV); (b) variable 28° (28V) versus variable 20° (20V); (c) variable 20° (20V) versus nonvariable 20° (20NV). *X*‐axis and *y*‐axis are expression values for the different treatments, measured as the log_2_ count of reads mapped to each transcript, normalized for the library size, averaged between replicates. Genes above the diagonal 1:1 line have higher expression in the *y*‐axis treatment, while genes below the diagonal line have higher expression in the *x*‐axis treatment

**Figure 3:**
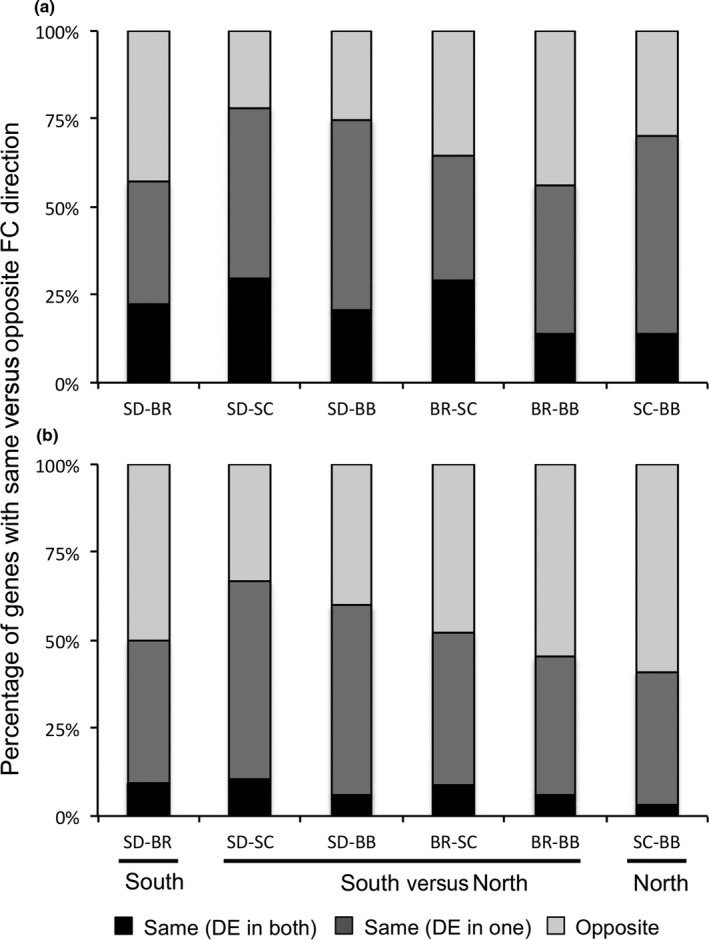
Percentage of genes with same versus opposite fold change direction in pairwise comparisons. (a) Stress at 28° (28ST) versus nonvariable 20° (20NV); (b) variable 28° (28V) versus variable 20° (20V). Fold change direction (but not magnitude) was determined for genes that were differentially expressed in one or both populations for all six pairwise comparisons. In both (a) and (b), within‐region comparisons do not have a higher percentage of genes changing in the same direction than the between‐region comparisons. Percentage of genes changing in the same direction is lower in the variable temperature regime for all comparisons. “Same (DE in both)” are genes that are differentially expressed in both populations, and fold change is in the same direction. “Same (DE in one)” are genes that are differentially expressed in only one of the two populations, but fold change is in the same direction in both. “Opposite” are genes with fold change in opposite direction in each of the populations

For the northern populations, BB‐N differentially expressed a higher number of genes than the two southern populations (204 up, 33 down; versus 100 up and 7 down for SD‐S, and 74 up and 11 down for BR‐S), while SC‐N had the lowest number of DE genes of the four populations in the 28ST versus 20NV comparison (52 up and 2 down; Figure 2a and Appendix S1). The composition of DE genes in the 28ST versus 20NV comparison indicates that each population has a mostly unique pattern of differential gene expression for this stress. Only 25 genes were DE in all four populations in this comparison (Table S2), and while the majority of these shared DE genes are well‐characterized genes involved in heat‐shock response (including 12 HSP genes), they only make up between 10 and 46% of all DE genes in each population. In pairwise comparisons, the populations share only a few more genes than these 25 common DE genes, but southern and northern populations do not share more DE genes within region than they do between regions (Figure 3a; Appendix S2). Even if we consider genes that are DE in at least one of the populations in pairwise comparisons and look at the direction of the expression change (regardless of whether or not the *p*‐value threshold is met), southern and northern populations share approximately the same percentage of genes within and between regions, with some between‐region comparisons sharing a higher percentage of genes that change in the same direction (Figure 3a; Appendix S2). The magnitude of the fold change of these genes, however, may be more informative. Overall, SD‐S on average upregulated the shared 25 DE genes to the smallest degree compared with the other populations, and this increased as you moved north, with BB‐N showing the highest average magnitude of change in expression of these genes (Table S2).

In all four populations, GO enrichment analysis found that terms associated with response to stress, unfolded protein binding, and protein folding (except for BB‐N) were enriched in DE genes that were upregulated (Table 1). Heat‐shock protein genes contributed to the enrichment of these terms and were the most common class of upregulated genes (16 in SD‐S, 13 in BR‐S, 14 in SC‐N, and 15 in BB‐N). GO terms related to protein targeting and cell motility were also enriched in SD‐S, while structural molecule activity and extracellular region terms were enriched in downregulated genes in BB‐S. In this last case, these terms are overrepresented in large part due to the large number of cuticle proteins that are DE (especially in BB‐N; Table 1).

**Table 1:** Enriched gene ontology (GO) terms for differentially expressed genes in the three treatment comparisons

Term	Category	*p* value	# Genes with term	Expression change	GO‐ID
28ST versus 20NV					
SD‐S					
Response to stress	P	1.47E−12	23	Up	GO:0006950
Unfolded protein binding	F	1.02E−07	7	Up	GO:0051082
Protein folding	P	2.97E−04	6	Up	GO:0006457
Protein targeting	P	6.05E−03	4	Up	GO:0006605
Cell motility	P	2.65E−02	4	Up	GO:0048870
BR‐S					
Response to stress	P	7.37E−08	16	Up	GO:0006950
Unfolded protein binding	F	7.54E−07	6	Up	GO:0051082
Protein folding	P	1.39E−03	5	Up	GO:0006457
SC‐N					
Response to stress	P	2.29E−11	17	Up	GO:0006950
Unfolded protein binding	F	6.26E−06	5	Up	GO:0051082
Protein folding	P	4.15E−03	4	Up	GO:0006457
BB‐N					
Response to stress	P	3.75E−08	26	Up	GO:0006950
Unfolded protein binding	F	1.93E−04	6	Up	GO:0051082
Structural molecule activity	F	0.00147	4	Down	GO:0005198
Extracellular region	C	0.02583	3	Down	GO:0005576
28V versus 20V					
SD‐S					
Structural molecule activity	F	1.73E−09	15	Up	GO:0005198
Response to stress	P	1.08E−08	18	Up	GO:0006950
Unfolded protein binding	F	1.44E−06	6	Up	GO:0051082
Extracellular region	C	3.68E−02	7	Up	GO:0005576
Protein folding	P	2.36E−03	5	Up	GO:0006457
Protein targeting	P	3.68E−02	3	Up	GO:0006605
BR‐S					
Unfolded protein binding	F	0.0301	3	Up	GO:0051082
Structural molecule activity	F	4.24E−03	5	Down	GO:0005198
Hydrolase activity, acting on carbon–nitrogen (but not peptide) bonds	F	4.24E−03	3	Down	GO:0016810
Carbohydrate metabolic process	P	2.08E−02	4	Down	GO:0005975
SC‐N					
Response to stress	P	3.35E−07	10	Up	GO:0006950
Unfolded protein binding	F	1.73E−03	2	Up	GO:0051082
BB‐N					
Response to stress	P	1.16E−06	17	Up	GO:0006950
Unfolded protein binding	F	2.60E−03	4	Up	GO:0051082
Structural molecule activity	F	3.79E−51	71	Down	GO:0005198
Extracellular region	C	4.69E−06	27	Down	GO:0005576
20V versus 20NV					
SD‐S					
Structural molecule activity	F	3.07E−13	21	Up	GO:0005198
Peptidase activity	F	2.94E−04	8	Down	GO:0008233
BR‐S					
Extracellular region	C	3.28E−03	6	Up	GO:0005576
Carbohydrate metabolic process	P	3.40E−02	4	Up	GO:0005975
Hydrolase activity	F	3.40E−02	8	Up	GO:0016787
Peptidase activity	F	4.64E−02	4	Down	GO:0008233
SC‐N					
Extracellular region	C	2.43E−12	78	Up	GO:0005576
Cell wall organization or biogenesis	P	2.50E−06	10	Up	GO:0071554
Carbohydrate metabolic process	P	4.10E−05	48	Up	GO:0005975
Structural molecule activity	F	2.45E−04	42	Up	GO:0005198
Hydrolase activity	F	1.73E−03	129	Up	GO:0016787
Oxidoreductase activity	F	6.66E−03	66	Up	GO:0016491
Sulfur compound metabolic process	P	2.39E−02	15	Up	GO:0006790
Cytoskeletal protein binding	F	2.42E−02	22	Up	GO:0008092
Nucleus	C	2.52E−06	100	Down	GO:0005634
Chromosome	C	7.13E−04	29	Down	GO:0005694
Cellular nitrogen compound metabolic process	P	2.26E−02	114	Down	GO:0034641
DNA binding	F	1.04E−02	44	Down	GO:0003677
Protein binding transcription factor activity	F	1.04E−02	11	Down	GO:0000988
Cell cycle	P	1.04E−02	32	Down	GO:0007049
External encapsulating structure	C	2.26E−02	3	Down	GO:0030312
Peptidase activity	F	3.07E−02	36	Down	GO:0008233
Microtubule organizing center	C	3.92E−02	11	Down	GO:0005815
BB‐N					
Structural molecule activity	F	6.46E−15	38	Up	GO:0005198
Hydrolase activity	F	1.90E−03	59	Down	GO:0016787
Extracellular region	C	3.59E−02	22	Down	GO:0005576
Oxidoreductase activity	F	3.59E−02	29	Down	GO:0016491
Carbohydrate metabolic process	P	3.59E−02	18	Down	GO:0005975
Lyase activity	F	3.59E−02	9	Down	GO:0016829

“Category” refers to the GO categories: C, cellular component; F, molecular function; P, biological process. *p* values are corrected for multiple comparisons using a false discovery rate of 5%.

SD‐S, San Diego; BR‐S, Bird Rock; SC‐N, Santa Cruz; BB‐N, Bodega Bay.

### 3.4 Response to moderate thermal stress in variable versus nonvariable temperature regimes (28ST vs. 20NV compared to 28V vs. 20V)

Next, we looked at the effects that raising individuals in a variable temperature regime (20°–28°) would have compared to the effects of raising them at a nonvariable 20° and exposing them to 28° as adults. Between 28V and 20V, BB‐N differentially expressed a larger number of genes than the southern populations (107 up, 384 down versus 94 up and 34 down for SD‐S, and 66 up and 34 down for BR‐S), but once again SC‐N had a much lower number of DE genes than the other populations (28 up, four down) (Figure 2b and Appendix S1). Only shared 10 genes were DE in all four populations in this comparison (Table S2), and as for 28ST versus 20NV, only a small number of DE genes were shared between pairs of populations (Figure 3b; Appendix S3). The differences in gene expression profile (measured as the percentage of genes with the same fold change direction in pairwise comparisons) between the populations were higher in the 28V versus 20V than in the 28ST versus 20NV comparison (Figure 3, Appendices S2 and S3).

**Figure 4:**
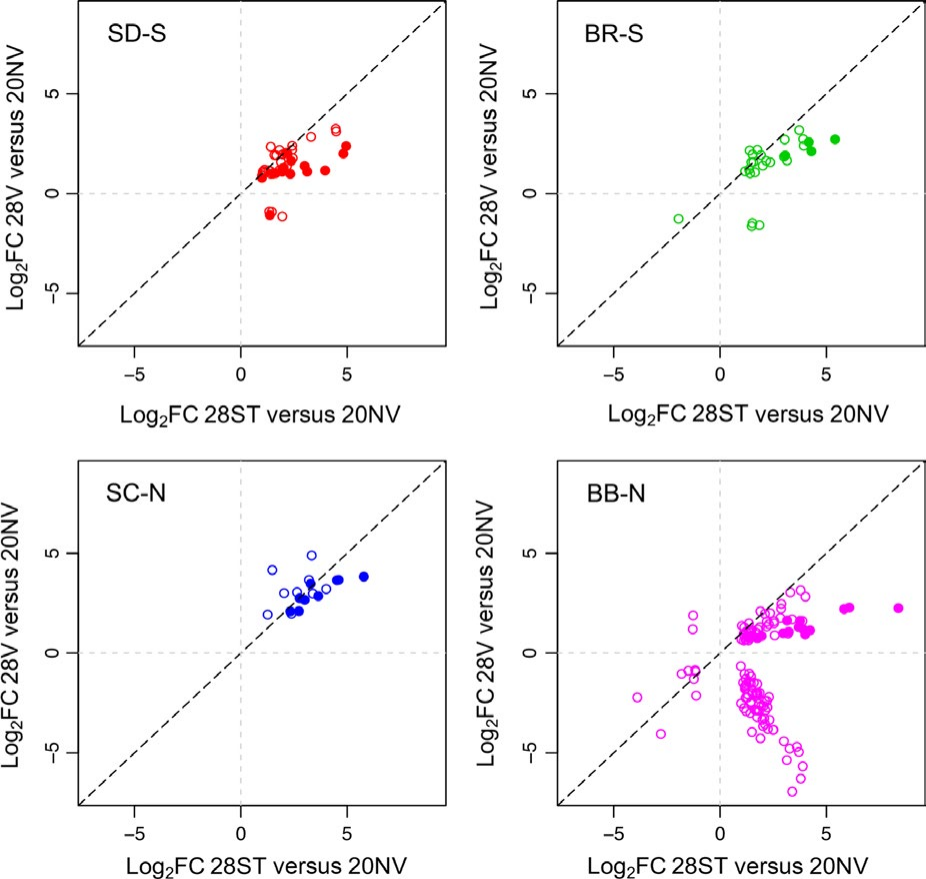
Comparison of log_2_ fold change (FC) for genes that are differentially expressed in both 28ST versus 20NV and 28V versus 20V. Filled circles are heat‐shock protein genes; open circles are all other genes. Diagonal black line is the 1:1 line; genes below this line have higher FC in **28ST versus 20NV**, and genes above the line have higher FC in **28V versus 20V**. Gray lines separate the four quadrants for up‐ and downregulated genes in the two comparisons

Differences between the 28ST versus 20NV and 28V versus 20V comparisons are due to phenotypic plasticity of gene expression, and if this plasticity is dampened when copepods experience a temperature on a daily basis (20°–28° variable regime), we would expect a smaller number of DE genes in all populations, and lower fold change for genes involved in heat‐shock response. Most genes that were DE in both 28ST versus 20NV and 28V versus 20V had lower fold change in the variable regime, consistent with our expectations (Figure 4). This was especially true for HSPs where in all but one case (an HSP 90 in SC‐N), the fold change was lower in 28V versus 20V. BB‐N showed the highest difference in fold change between 28ST versus 20NV and 28V versus 20V, indicating it is expressing the greatest degree of plasticity in gene expression, with different HSPs 70 and 90 going from a range of 333.38‐8.88 fold change in 28ST versus 20NV to 4.85‐2.30 fold change in 28V versus 20V. Several other genes went from being upregulated in 28ST versus 20NV to downregulated in 28V versus 20V, including several cuticle proteins and lectins (Figure 4; Appendix S1). Even though SC‐N had a much smaller number of DE genes than any of the other populations, the magnitude of the potentially plastic gene expression change was high for several genes. For example, fold change between three HSP 70 genes (TCALIF_06728, TCALIF_04517, comp38417) decreased from 54.94, 24.16, and 22.90, to 14.18, 12.69, and 12.55, respectively (Appendix S1). However, even though the fold change decreased in the variable regime, fold change of these HSP genes is the highest for any population in this regime. Southern populations also showed plastic responses with HSP 70 and 90 genes showing fold change decreasing in SD‐S from a range of 30.90–4.59 in 28ST versus 20NV to 5.19–1.97 in 28V versus 20V, and in BR‐S from a range of 42.41–8.34 in 28ST versus 20NV to 6.55–3.75 in 20V‐c28 (Figure 4; Appendix S1).

GO terms that were enriched in the 28V versus 20V comparison included response to stress and unfolded protein binding for upregulated genes in SD‐S, SC‐N, and BB‐N, while BR‐S only had the unfolded protein biding term enriched. These two terms were enriched in all populations for the 28ST versus 20NV comparison as well. Terms associated with structural molecule activity and extracellular region were also enriched in upregulated genes in SD‐S, and downregulated genes in BB‐N. BR‐S had enrichment of structural molecule activity on downregulated genes as well (Table 1). As mentioned before, cuticle protein genes contribute to this enrichment.

### 3.5 Differential expression between thermal regimes (20V versus 20NV)

Lastly, we looked at how the levels of gene expression differed when the populations were at 20° in the nonvariable versus the variable regimes (20V vs. 20NV). Genes that were upregulated in this comparison had higher expression in 20V, while downregulated genes had higher expression in 20NV. Unlike the two previous comparisons, 20V versus 20NV displays the biggest difference in the pattern of gene expression between southern and northern populations, where both northern populations differentially expressed a much larger number of genes (both up and down) than southern populations (Figure 2c and Appendix S1), and SC‐N differentially expressed many more genes than any of the other populations (BB‐N: 437 up, 578 down; SC‐N: 1369 up, 720 down; BR: 45 up, 38 down; SD‐S: 172 up, 52 down). Within southern populations, SD‐S differentially expressed more genes in both directions than BR; however, it did so to a lower magnitude, especially in upregulated genes (SD‐S average fold change: 4.48 up‐ and 2.10 downregulated; BR‐S average fold change 10.95 up‐ and 2.22 downregulated; Appendix S1).

The number of DE genes in northern populations was between 4.5 and 25 times greater than in southern populations for this comparison. However, even though the number of DE genes was more similar within a region than between regions, the actual set of genes and their functions were very different for each population. GO enrichment analysis (Table 1) found structural molecule activity genes to be enriched in upregulated genes in SD‐S, while peptidase activity genes were enriched in downregulated genes in these populations. In BR‐S, extracellular region, carbohydrate metabolic process, and hydrolase activity terms were enriched in upregulated genes, and peptidase activity terms are enriched in downregulated genes. In SC‐N, eight terms were enriched for upregulated genes, while nine were enriched for downregulated genes (Table 1). Several of these enriched GO terms in downregulated genes indicate that SC‐N has to downregulate a number of genes that are involved in basic cell maintenance processes. In BB‐N, genes associated with structural molecule activity are overrepresented in upregulated genes, while three catalytic activity terms, as well as, extracellular region, and carbohydrate metabolic process genes are enriched in downregulated genes (Table 1).

One reason for genes to be upregulated at 20V compared to 20NV could be in “anticipation” of the higher temperatures individuals in the variable regime experience daily compared to those in the nonvariable regime (frontloading). In this study, these frontloaded genes would be genes that were upregulated in 28ST versus 20NV as well as in 20V versus 20NV. SD‐S had 18 frontloaded genes (including six HSPs and one cuticle protein), BR‐S had 10 frontloaded genes (including one small HSP and two cuticle proteins), SC‐N had four frontloaded genes (three HSPs), and BB‐N had 84 frontloaded genes (including eight HSPs and seven cuticle proteins) (Appendix S1).

## 4 Discussion

We used RNA‐seq to determine transcriptome‐wide patterns of gene expression for locally adapted populations of the copepod *T. californicus* in two thermal regimes. The higher temperature experienced by these populations (28°) should present a moderate stress, but all of the populations encounter this temperature in nature (Kelly et al., 2012), and it is well below their acute lethal temperature (Kelly et al., 2012; Pereira et al., 2014; Tangwancharoen & Burton, 2014; Willett, 2010). Unlike studies that expose organisms to an acute thermal stress, the changes in gene expression we expect to observe are not the maximum response these organisms can mount, but the level of their response (both as the number of DE genes, as well as the magnitude of the expression change) should indicate the level of thermal stress each population is experiencing. Our results can also give us insights into the differences in fitness that have been observed between these populations under these specific temperature regimes (Willett, 2010).

### 4.1 Transcriptome‐wide response to moderate thermal stress differs between populations

The most striking result in this study was the level of differentiation in gene expression observed between the populations when they were exposed to a moderate heat stress (28°). This was particularly surprising for the two southern populations (SD‐S and BR‐S), which are separated by only 8 km and should share roughly similar thermal environments overall. There are, however, aspects of the biology of these copepods that can explain our results. There is extremely low gene flow between these two populations (Burton, 1997; Willett & Ladner, 2009), and shared polymorphism is also very low. Pereira et al. (2016) showed that shared polymorphism rapidly decreases as divergence increases between populations of this species. Mitochondrial DNA divergence is ~10% between SD‐S and BR‐S, and it can be >20% between the southern and northern populations used in this study (Burton, 1998; Willett & Ladner, 2009). Our results may also help partially explain the observed transgressive segregation that has been shown in hybrids between the SD‐S and BR‐S populations (Pereira et al., 2014). Late‐generation hybrids (F9) between these two populations have higher thermal tolerance than either parental population, and some hybrid lines were even able to survive temperatures that are lethal to both parental populations. The results presented would suggest that this increased thermal tolerance of hybrids could be due to these two populations having evolved different ways to deal with increases in temperatures. When the two genomes are combined in hybrids, complementary gene action of factors that are associated with higher heat tolerance could then lead to transgressive, higher tolerance in these hybrids; however, this remains to be tested.

When we consider the two northern populations, where differences in gene expression profiles were even more dramatic, a combination of differences in abiotic selective pressures and population history is likely to play a more important role than between the southern populations. These northern populations are approximately 240 km apart and are twice as genetically divergent as the southern populations (Figure 1a; Pereira et al., 2016). It is important to remember, however, that these two populations have similar upper thermal limits (Willett, 2010), have similar thermal performance curves (Hong & Shurin, 2015), and are both outcompeted by the southern populations when raised in variable 20°–28° environment (Willett, 2010). Therefore, these thermal phenotypes might have different genetic underpinnings between these populations. While it is not uncommon for species or populations that differ in thermal tolerance to show different sets of DE genes with small overlap between them (Barshis et al., 2013; Dayan et al., 2015; Franssen et al., 2014; Gleason & Burton, 2015; Narum & Campbell, 2015; Wang et al., 2014), our results are unique as we observe large differences between populations of the same species with similar thermal tolerance phenotypes.

### 4.2 Possible genetic underpinnings for lower competitiveness of northern populations

Willett (2010) showed that at the same variable temperature regime used here, southern populations outcompete northern ones. Our results elucidate some of the possible gene expression patterns that contribute to this difference in competitiveness. The major pattern is that the southern populations, which are better adapted to warm temperatures, have to change the expression of a much smaller number of genes and do so to lower levels (lower fold change). But maybe more surprising is the fact that very different gene expression patterns are associated with the lower relative fitness of the northern populations at these temperatures. BB‐N displayed what we considered to be an expected response to a moderate thermal stress, differentially expressing a large number of genes between 28° and 20° in both thermal regimes, with high levels of fold change compared to the other populations (especially for HSP genes). This, however, was not the case for SC‐N, as it did not change the expression of a large number of genes between 28° and 20°, and even the magnitude of change for DE genes was not much higher than that of the southern populations (except for a small number of HSP genes). We suggest that the lower competitive ability of SC‐N compared to SD‐S in the variable temperature regime (Willett, 2010) may then be due to how it responds to the high‐temperature variable regime, compared to the nonvariable regime, and not specifically because of how it responds to a temperature change from 20° to 28°. It is important to mention that SC‐N outcompetes SD‐S at a low‐temperature variable regime (16°–25°), so it is the combination of variable and high temperatures that leads to the observed pattern. SC‐N is maintaining a large number of genes (including some HSPs) at higher levels of expression at both 20V and 28V than at 20NV, while downregulating a large number of genes that are important for basic cellular processes at both temperatures in the variable regime (Table 1; Appendix S1). Therefore, it is possible that SC‐N may have lower fitness at these variable temperatures not solely due to their level of heat‐shock response when exposed to moderately stressful temperatures on a regular basis, as appears to be the case for BB‐N, but also potentially because of how much it has to change its metabolic framework as a whole when it lives in these variable temperatures.

### 4.3 General patterns of gene expression across all populations

It is interesting that even at this moderately high temperature (28°), all populations display some level of heat‐shock response, upregulating a number of HSP genes and other genes associated with protein biding and refolding (Table 1, Appendix S1). This suggests that all populations, but especially the southern ones, often have to elicit a heat‐shock response during the warmer months in nature (Kelly et al., 2012). The production of HSPs, and their dependence on ATP for function (HSP 70 and 90), can add considerable ATP demand to the cell and negatively affect the organism (Clare & Saibil, 2013; Feder et al., 1992; King & MacRae, 2015; Tomanek, 2010). Therefore, even though this heat‐shock response is likely an adaptation that allows these organisms to persist when exposed to these temperatures, increases in mean temperature due to climate change may have a strong negative impact in this species. The southern populations already experience these temperatures (or higher) often in nature, and as is the case for many other intertidal species (Tomanek, 2010), increases in their ambient temperature would mean they have to mount this heat‐shock response for a higher proportion of time, possibly leading to decreased fitness.

As was observed in a previous RNA‐seq study in *T. californicus* (Schoville et al., 2012), a number of genes that are annotated as cuticle protein genes are DE in all populations and are overrepresented in at least one of the treatments comparisons, although the direction of the expression change differs between the populations (Table 1). While we do not know the exact function these genes/proteins have in response to thermal stress, cuticle protein genes have been observed to be DE in studies of thermal adaptation in *Drosophila* (Zhao et al., 2015), and a gene homologous to a DE *T. californicus* cuticle protein gene (Contig_59_58) in *Anopheles gambiae* (agap006369‐pa) is annotated with a GO term associated with stress response. This gene occurs in *A. gambiae* within an inversion that contains a large cluster of cuticle protein genes, as well as three *hsp83* genes (White et al., 2007); however, it is still unknown the role these cuticle protein genes play in heat or desiccation resistance in this mosquito. While we do not know the function these genes are serving, it is possible that cuticle‐associated proteins are part of the thermal response in arthropods.

Studies in *Chlorostoma* snails, a species of *Acropora* corals, and two species of seagrass show that more thermally tolerant populations or species in these groups have higher constitutive levels of HSP gene expression (frontloading), which may enable them to more readily respond to thermal stress (Barshis et al., 2013; Dong et al., 2010; Franssen et al., 2014; Gleason & Burton, 2015; Tomanek & Somero, 1999). The same may be expected in individuals that were raised in a variable environment compared to a nonvariable one, where some genes that respond to thermal stress in the nonvariable temperature environment (28ST versus 20NV) would also be upregulated at 20° in the variable environment compared to 20° in the nonvariable environment (20NV‐20V). Frontloading genes can be seen as a form of plastic response to higher daily temperatures, and while all populations have at least some frontloaded genes, BB‐N has by far the most, including not only several HSP genes, but also several cuticle protein and lectin genes (Appendix S1). SC‐N has a small number of upregulated genes at 20NV‐28ST, which limits the number of frontloaded genes it can have in this case, but of the four genes that are frontloaded, three are HSP genes (Appendix S1). The two southern populations have intermediate numbers of frontloaded genes (18 for SD‐S and 10 for BR‐S), again suggesting that the two northern populations mount very different molecular responses to the variable temperature environment.

The present study determined a small set of candidate genes that are likely to be the product of adaptive plastic response to the different temperature treatments. These are the genes that are DE in all populations in the different treatment comparisons: 25 DE genes for 28ST versus 20NV, and 10 for 28V versus 20V (seven common to both comparisons; Table S2). The large majority of these genes are associated with heat‐shock response, with a strong overrepresentation of HSP genes (Table S2). One of these genes codes for *hsp beta‐1*, a gene on which Barreto, Schoville, and Burton (2015) performed RNAi using *T. californicus*, and showed that individual copepods that had this gene knocked down had lower thermal tolerance compared to control individuals. In the present study, this gene is not only upregulated in all four populations at 28° in both thermal regimes, but it also shows strong signs of plasticity with a dampening of gene expression in comparisons of nonvariable and variable temperature environment in all but one population; SC‐N has very similar levels of fold change between 28° and 20° in both thermal regimes. While we do not have direct evidence that the other genes in this group are the result of adaptive plastic response, they are strong candidates for further work into their function in thermal stress response.

## 5 Conclusion

The present study highlights some key ways that local adaptation can impact the manner in which an organism deals with temperature changes at the level of gene expression. First, it is clear that locally adapted populations of the same species can display different responses at the molecular level despite showing similar thermal performance phenotypes. This was particularly striking between SD‐S and BR‐S, which are only 8 km apart and yet show substantial differences in their responses to both temperature variability and moderate heat stress. Therefore, even for closely related populations, the molecular mechanisms they use to deal with temperature changes can be different. Second, as seen in the northern populations, the molecular response to changes in thermal variability (i.e., 20NV vs. 20V comparison) may be drastically different, even though their upper thermal limit is very similar. Therefore, we may be underestimating the amount of variation to stress response at the molecular, and potentially physiological, level in species with very segregated populations, by assuming that populations with similar thermal tolerances respond to changes in temperature in the same way.

## Conflict of Interest

None declared.
