# Supplementary material for: Locally adapted populations of a copepod can evolve different gene expression patterns under the same environmental pressures
Source: Ecol Evol. 2017 May 9;7(12):4312–25. doi: 10.1002/ece3.3016 (PMC5478056; doi:10.1002/ece3.3016)
Supplement: Supplementary file 1 [file ECE3-7-4312-s001.docx]

**Figure S1 Experimental design.** Individuals from all populations were exposed to two thermal regimes (non-variable and variable) from birth through adulthood. RNA was isolated from both regimes at 20˚ at the end of the 20˚ portion of the variable regime. RNA was isolated from both regimes at 28˚, after two hours at this temperature.
