# Supplementary material for: Locally adapted populations of a copepod can evolve different gene expression patterns under the same environmental pressures
Source: Ecol Evol. 2017 May 9;7(12):4312–25. doi: 10.1002/ece3.3016 (PMC5478056; doi:10.1002/ece3.3016)
Supplement: Supplementary file 2 [file ECE3-7-4312-s002.docx]

**Figure S2**. Multidimensional scaling (MDS) plots showing the level of similarity between each RNA sample. **a.** All samples for all populations. Samples clustered by population and not by treatment. Dimension 1 separates southern and northern populations, as well as the two northern populations. **b.** RNA samples from SD-S. **c.** RNA samples from BR-S. **d.** RNA samples from SC-N. **e.** RNA samples from BB-N. Population specific MDS plots, show that many cases RNA samples that were sequenced in the same Illumina lane are more similar than they are to their replicate. This indicates a batch effect in the samples. To deal with this a generalized linear model was fit to the data, and the sample “Illumina lane” was used as a blocking factor.
