# Supplementary material for: Locally adapted populations of a copepod can evolve different gene expression patterns under the same environmental pressures
Source: Ecol Evol. 2017 May 9;7(12):4312–25. doi: 10.1002/ece3.3016 (PMC5478056; doi:10.1002/ece3.3016)
Supplement: Supplementary file 3 [file ECE3-7-4312-s003.docx]

**Table 1.** Number of reads for each sample after trimming, and the number of reads and percentages that were mapped to the references.

| **Treatment** | **Trimmed reads** | **Mapped to GT** | **Mapped to DNT** | **Total mapped** | **% mapped** |
| --- | --- | --- | --- | --- | --- |
| **SD-S** | | | | | |
| 20NV-1 | 12,872,554 | 4,555,835 | 3,543,925 | 8,099,760 | 62.92 |
| 20NV-2 | 17,143,308 | 8,474,716 | 4,879,581 | 13,354,297 | 77.90 |
| 28ST-1 | 16,408,726 | 5,664,363 | 4,729,381 | 10,393,744 | 63.34 |
| 28ST-2 | 17,179,316 | 8,178,326 | 5,228,211 | 13,406,537 | 78.04 |
| 20V-1 | 14,355,582 | 5,145,499 | 4,020,190 | 9,165,689 | 63.85 |
| 20V-2 | 17,083,468 | 8,095,867 | 5,083,644 | 13,179,511 | 77.15 |
| 28V-1 | 11,892,344 | 4,099,376 | 3,476,360 | 7,575,736 | 63.70 |
| 28V-2 | 20,240,034 | 11,182,234 | 5,004,946 | 16,187,180 | 79.98 |
| **BR-S** | | | | | |
| 20NV-1 | 9,352,537 | 2,955,978 | 2,361,620 | 5,317,598 | 56.86 |
| 20NV-2 | 22,293,237 | 9,983,751 | 7,060,271 | 17,044,022 | 76.45 |
| 28ST-1 | 16,225,920 | 5,396,336 | 3,798,957 | 9,195,293 | 56.67 |
| 28ST-2 | 21,841,078 | 9,858,588 | 6,955,168 | 16,813,756 | 76.98 |
| 20V-1 | 7,698,444 | 2,419,610 | 1,956,037 | 4,375,647 | 56.84 |
| 20V-2 | 12,793,717 | 5,672,030 | 3,853,155 | 9,525,185 | 74.45 |
| 28V-1 | 11,750,350 | 3,596,306 | 3,207,364 | 6,803,670 | 57.90 |
| 28V-2 | 29,073,232 | 10,074,266 | 5,208,716 | 15,282,982 | 52.57 |
| **SC-N** | | | | | |
| 20NV-1 | 11,428,402 | 3,705,020 | 3,275,656 | 6,980,676 | 61.08 |
| 20NV-2 | 16,766,684 | 6,963,207 | 6,079,277 | 13,042,484 | 77.79 |
| 28ST-1 | 10,208,731 | 3,300,905 | 2,865,960 | 6,166,865 | 60.41 |
| 28ST-2 | 10,532,089 | 4,441,460 | 3,686,869 | 8,128,329 | 77.18 |
| 20V-1 | 11,688,633 | 3,545,760 | 3,431,916 | 6,977,676 | 59.70 |
| 20V-2 | 17,887,164 | 7,834,548 | 5,695,801 | 13,530,349 | 75.64 |
| 28V-1 | 11,179,377 | 4,083,251 | 2,397,768 | 6,481,019 | 57.97 |
| 28V-2 | 23,762,493 | 10,456,973 | 7,434,340 | 17,891,313 | 75.29 |
| **BB-N** | | | | | |
| 20NV-1 | 13,127,029 | 4,179,023 | 3,535,189 | 7,714,212 | 58.77 |
| 20NV-2 | 18,095,437 | 8,154,582 | 6,013,711 | 14,168,293 | 78.30 |
| 28ST-1 | 8,567,321 | 2,776,597 | 2,182,853 | 4,959,450 | 57.89 |
| 28ST-2 | 12,944,412 | 5,779,006 | 4,327,404 | 10,106,410 | 78.08 |
| 20V-1 | 11,378,699 | 3,750,432 | 2,845,145 | 6,595,577 | 57.96 |
| 20V-2 | 15,814,360 | 7,492,404 | 4,820,856 | 12,313,260 | 77.86 |
| 28V-1 | 13,509,632 | 4,475,930 | 3,469,773 | 7,945,703 | 58.82 |
| 28V-2 | 14,105,530 | 6,780,762 | 4,245,735 | 11,026,497 | 78.17 |

Note – SD-S, San Diego; BR-S, Bird Rock; SC-N, Santa Cruz; BB-N, Bodega Bay.
