# Supplementary material for: Locally adapted populations of a copepod can evolve different gene expression patterns under the same environmental pressures
Source: Ecol Evol. 2017 May 9;7(12):4312–25. doi: 10.1002/ece3.3016 (PMC5478056; doi:10.1002/ece3.3016)
Supplement: Supplementary file 4 [file ECE3-7-4312-s004.docx]

**Table S2.** Genes that are differentially expressed in all four populations in the constant temperature regime (28ST vs 20NV), and the variable temperature regime (28V vs 20V). Fold change refers to the expression at 28˚ compared to 20˚ within each population in each regime. Bold indicates genes that are differentially expressed in both regimes.

| **Gene ID** | **Description** | **Fold Change** | | | |
| --- | --- | --- | --- | --- | --- |
|  |  | **SD** | **BR** | **SC** | **BB** |
| **28ST vs 20NV** | | | | | |
|  | | | | | |
| **comp51313_c0_seq1_44507** | **N/A** | **9.85** | **13.18** | **9.21** | **10.42** |
| Contig20_19 | N/A | 4.32 | 3.37 | 4.41 | 5.1 |
| TCALIF_03375 | N/A | 22.3 | 29.35 | 16.15 | 16.14 |
| **TCALIF_03376** | **N/A** | **21.99** | **14.99** | **10.44** | **14.61** |
| TCALIF_00753 | 78 kda glucose-regulated partial | 3.89 | 5.13 | 5.67 | 4.91 |
| comp32704_c0_seq2_17919 | 78 kda glucose-regulated protein precursor | 3.69 | 5.27 | 5.2 | 4.57 |
| TCALIF_01534 | a chain orally active 2-amino thienopyrimidine inhibitors of the hsp90 chaperone | 4.49 | 8.04 | 5.44 | 6.86 |
| TCALIF_01814 | adhesion lipoprotein | 5.24 | 4.02 | 5.14 | 7.37 |
| TCALIF_09395 | bag domain-containing protein samui-like isoform x3 | 2.53 | 2.79 | 3.68 | 5.93 |
| **TCALIF_02439** | **cd63 antigen** | **5.33** | **4.49** | **6.25** | **7.11** |
| TCALIF_05614 | heat shock protein 40 | 2.22 | 2.83 | 2.48 | 3.88 |
| **TCALIF_04517** | **heat shock protein 70** | **30.9** | **42.41** | **22.9** | **67.36** |
| **TCALIF_06728** | **heat shock protein 70** | **28.22** | **19.63** | **54.94** | **333.38** |
| TCALIF_09482 | heat shock protein 70 | 4.59 | 2.79 | 5.48 | 8.88 |
| **comp46685_c0_seq1_33583** | **heat shock protein 90** | **5.14** | **8.34** | **9.72** | **14.89** |
| TCALIF_07011 | heat shock protein beta-1 | 3.1 | 2.68 | 3.34 | 3.94 |
| **TCALIF_10081** | **heat shock protein beta-1** | **15.59** | **18.03** | **6.78** | **18.74** |
| TCALIF_13714 | heat shock protein beta-1 | 3.85 | 4.4 | 6.15 | 7.8 |
| TCALIF_13715 | heat shock protein beta-1 | 4.05 | 4.96 | 12.47 | 12.95 |
| TCALIF_04918 | heat shock protein hsp16- | 4.74 | 4.22 | 5 | 9.13 |
| TCALIF_00957 | protein isoform b | 2.72 | 1.96 | 2.85 | 5.14 |
| TCALIF_06394 | small heat shock protein | 6.8 | 6.21 | 6.35 | 10.42 |
| TCALIF_13523 | small heat shock protein | 4.25 | 5.63 | 6.63 | 9.39 |
| TCALIF_05480 | unkown protein | 2.68 | 2.83 | 3.48 | 4.55 |
| TCALIF_09115 | x-box binding protein 1 | 3.52 | 3.11 | 5.12 | 5.81 |
| **28V vs 20V** | | | | | |
| **comp51313_c0_seq1_44507** | **---NA---** | **7.18** | **9.06** | **12.65** | **3.23** |
| TCALIF_00461 | ---NA--- | 3.77 | 5.66 | 8.01 | 8.23 |
| **TCALIF_03376** | **---NA---** | **9.46** | **6.61** | **7.87** | **3.05** |
| **TCALIF_02439** | **cd63 antigen** | **5.24** | **3.12** | **8.28** | **3.40** |
| **TCALIF_04517** | **heat shock protein 70** | **5.19** | **6.55** | **12.55** | **4.85** |
| **TCALIF_06728** | **heat shock protein 70** | **3.99** | **4.33** | **14.18** | **4.75** |
| **comp46685_c0_seq1_33583** | **heat shock protein 90** | **3.10** | **3.76** | **11.08** | **2.30** |
| **TCALIF_10081** | **heat shock protein beta-1** | **2.23** | **5.96** | **6.73** | **2.20** |
| TCALIF_06172 | hypothetical protein | 18.80 | -3.29 | 22.13 | -1.85 |
| TCALIF_13893 | short-chain dehydrogenase | 2.37 | 1.96 | 3.79 | 2.05 |

Note – SD-S, San Diego; BR-S, Bird Rock; SC-N, Santa Cruz; BB-N, Bodega Bay.
